# Metabolic syndrome and transaminases: systematic review and meta-analysis

**DOI:** 10.1186/s13098-023-01200-z

**Published:** 2023-10-30

**Authors:** Elena Raya-Cano, Rafael Molina-Luque, Manuel Vaquero-Abellán, Guillermo Molina-Recio, Rocío Jiménez-Mérida, Manuel Romero-Saldaña

**Affiliations:** 1 Faculty of Medicine and Nursing, University of Córdoba, Avd. Menéndez Pidal N/N, Córdoba, 14004 Spain; 2 Lifestyles, Innovation and Health (GA-16), Maimonides Biomedical Research Institute of Cordoba (IMIBIC), Córdoba, Spain

**Keywords:** Metabolic syndrome, Alanine transaminase, Aspartate aminotransferase, Gamma-glutamyltransferase, Biologic marker

## Abstract

**Background:**

Metabolic syndrome (MetS) is a group of metabolic abnormalities characterised by hypertension, central obesity, dyslipidaemia and dysregulation of blood glucose, associated with the risk of diabetes, cardiovascular disease and overall mortality. The presence of elevated liver enzymes may precede the development of MetS, with alterations of the liver being observed that are directly related to metabolic problems. The study aims to provide the best evidence on the association between liver enzymes (ALT, AST, GGT) and MetS by determining the effect size of these biomarkers.

**Methods:**

A systematic review and meta-analysis of studies indexed in PubMed and Scopus databases were performed. Study quality was assessed using the STROBE tool. The Grade Pro tool was used to evaluate the evidence, and the quantitative synthesis was performed using RevMan (Cochrane Collaboration).

**Results:**

Seventeen articles comparing liver enzyme concentrations between 76,686 with MetS (MetS+) and 201,855 without MetS (MetS-) subjects were included. The concentration of ALT, AST and GGT in the MetS + subjects was significantly higher than in the control group 7.13 IU/L (CI95% 5.73–8.54; p < 0.00001; I^2^ = 96%), 2.68 IU/L (CI95% 1.82–3.54; p < 0.00001; I^2^ = 96%) and 11.20 IU/L (CI95% 7.11–15.29; p < 0.00001; I^2^ = 96%), respectively.

**Conclusions:**

The evaluation of the relationship of liver enzymes in the pathophysiological process of MetS could lead to new insights into early diagnosis.

**Supplementary Information:**

The online version contains supplementary material available at 10.1186/s13098-023-01200-z.

## Background

Metabolic syndrome (MetS) encompasses several cardiovascular risk factors, including insulin resistance, atherogenic dyslipidaemia, central obesity and hypertension [1]. It is a multifactorial non-communicable disease that significantly contributes to morbidity and mortality and is considered a public health burden worldwide [2].

In addition to increasing the risk of cardiovascular disease (CVD), MetS and its risk factors, including obesity and diabetes mellitus (DM), are associated with liver disease. Liver function is essential for glucose and fatty acid metabolism. Hepatic glucose homeostasis influences insulin sensitivity, while peripheral insulin resistance and lipolysis contribute to fat accumulation in the liver (hepatic steatosis) [3].

In this regard, MetS has a direct relationship with non-alcoholic fatty liver disease (NAFLD) [4], both being predictors of the development of fibrosis and hepatocellular carcinogenesis [5].

NAFLD affects approximately 25% of the world’s population and is a leading cause of cirrhosis, hepatocellular carcinoma and liver transplantation [6]. This disorder, characterised by lipid deposition in hepatocytes, encompasses a group of liver diseases that resemble alcoholic liver disease, ranging from simple steatosis to steatohepatitis and cirrhosis [7]. These liver diseases have become the leading causes of liver-related morbidity and mortality and a risk factor for DM, chronic kidney disease, hypertension, MetS and CVD [8].

In this context, early liver impairment detection would help prevent or diagnose other metabolic disorders. According to recent studies, liver function tests, including serum alanine transaminase (ALT), aspartate transaminase (AST), alkaline phosphatase (ALP) and gamma-glutamyltransferase (GGT), can be valuable parameters in the assessment of metabolic status, especially in the investigation of cardio-metabolic disorders [9]. Specifically, several authors have explored the associations between liver enzymes, MetS, and CVD in different populations [10, 11]. In this regard, elevated ALT levels have been shown to help predict CVD in prospective studies [12, 13], and MetS and its components [14]. Although GGT is considered an indicator of the degree of liver disease and alcohol consumption, several studies have shown that the level of this enzyme is also associated with diabetes, hypertension and cardiovascular mortality independently of liver damage or alcohol consumption [15, 16]. One of the advantages of these parameters is that they are commonly measured in liver function tests and are well-known markers of liver damage [17].

Therefore, this possible relationship between serum liver enzymes and MetS has recently attracted much attention. Therefore, the main objective of the systematic review and meta-analysis is to provide the best degree of evidence on the association between liver enzymes (ALT, AST, GGT) and MetS, determining the effect size of these biomarkers.

## Methods

### Search strategy and eligibility criteria

This systematic review and meta-analysis were conducted according to the criteria established by the Preferred Reporting Items for Systematic Reviews and Meta-Analyses (PRISMA) [18] (Supplementary file). The search was carried out in the PubMed and Scopus databases. The search strategy was developed by combining the following Medical Subject Headings (MeSH) descriptors: (“aspartate aminotransferase” OR “alanine aminotransferase” OR “gamma-glutamyltransferase”) AND (“metabolic syndrome”) (Supplementary file). In addition, we included cross-sectional and longitudinal studies published between January 2017 and July 2022 that investigated the association between liver enzymes (ALT, AST, GGT) and MetS. In addition, the results had to include the mean and standard deviation. Only papers written in English and Spanish, and those that collected data from subjects over 18 years of age, were considered. The systematic review was registered in PROSPERO with ID CRD42023366810.

### Selection of papers

Two researchers (E.R.C and M.R.S) reviewed titles, abstracts and full texts. In addition, three researchers independently extracted data for studies that met the inclusion criteria (R.J.M, R.M.L. and G.M.R.). Finally, a fourth author (M.V.A.) acted as a judge in case of discrepancy. After sensitivity analysis, two articles [19, 20] were eliminated from the qualitative synthesis due to the heterogeneity of the reported data.

### Data extraction

One researcher (E.R.C.) was responsible for extracting the data verified by a second researcher (R.J.M.). A third researcher (M.R.S.) resolved the disagreement in case of a tie. Cohen’s Kappa index was used to assess the degree of agreement. The following data were extracted from each study: citation, details of the study population (including age and sex), study design, follow-up period, sample size, and mean and standard deviation of ALT, AST, and GGT in those subjects with Metabolic Syndrome (MetS+) and without Metabolic Syndrome (MetS-). In addition, for articles collecting ALT, AST and GGT data, the mean and standard deviation were extracted.

### Evaluation of the qualitative synthesis

The evaluation of the qualitative synthesis was carried out through a triple analysis, and four authors were responsible (R.M.L., R.J.M., E.R.C. and GMR):

- Assessment of methodological quality. The STROBE (Strengthening the Reporting of Observational Studies in Epidemiology) statement [21] was used for observational studies.
- Risk of bias assessment. Using the Cochrane Collaboration tool [22] included in the REVMAN 5.4.2 software, the risks of selection, conduct, detection, attrition and reporting were analysed.
- Assessment of the quality of evidence. With the help of the Grade Pro tool, the evidence profile table was developed, establishing the following levels [23]:
  High: high confidence in the match between the actual and estimated effect.
  Moderate: Moderate confidence in the effect estimate. There is a possibility that the actual effect is far from the estimated effect.
  Low: limited confidence in the estimate of the effect. The actual effect may be far from the estimated effect.
  Very low: low confidence in the estimated effect. The actual effect is very likely to be different from the estimated effect.

### Statistical analysis (evaluation of quantitative synthesis or meta-analysis)

The Cochrane Review Manager software (RevMan 5.4.2) was used for the meta-analysis to perform the statistical calculation and create the forest and funnel plots. Due to the difference in effect size of the included studies, a meta-analysis was performed using the Mantel-Haenszel random-effects method according to the DerSimonian and Laird model. The difference between arithmetic means with a 95% confidence interval was used to measure effect size. Liver enzyme counts were considered in IU/L. The risk of publication bias was assessed using the funnel plot. Heterogeneity was analysed using the Chi-square test and the inconsistency index (I^2^). According to the Cochrane Collaboration tool, heterogeneity was classified as follows: unimportant (0–40%), moderate (30–60%), substantial (50–90%) and considerable (75–100%).

## Results

### Characteristics of the studies

**Fig. 1:**
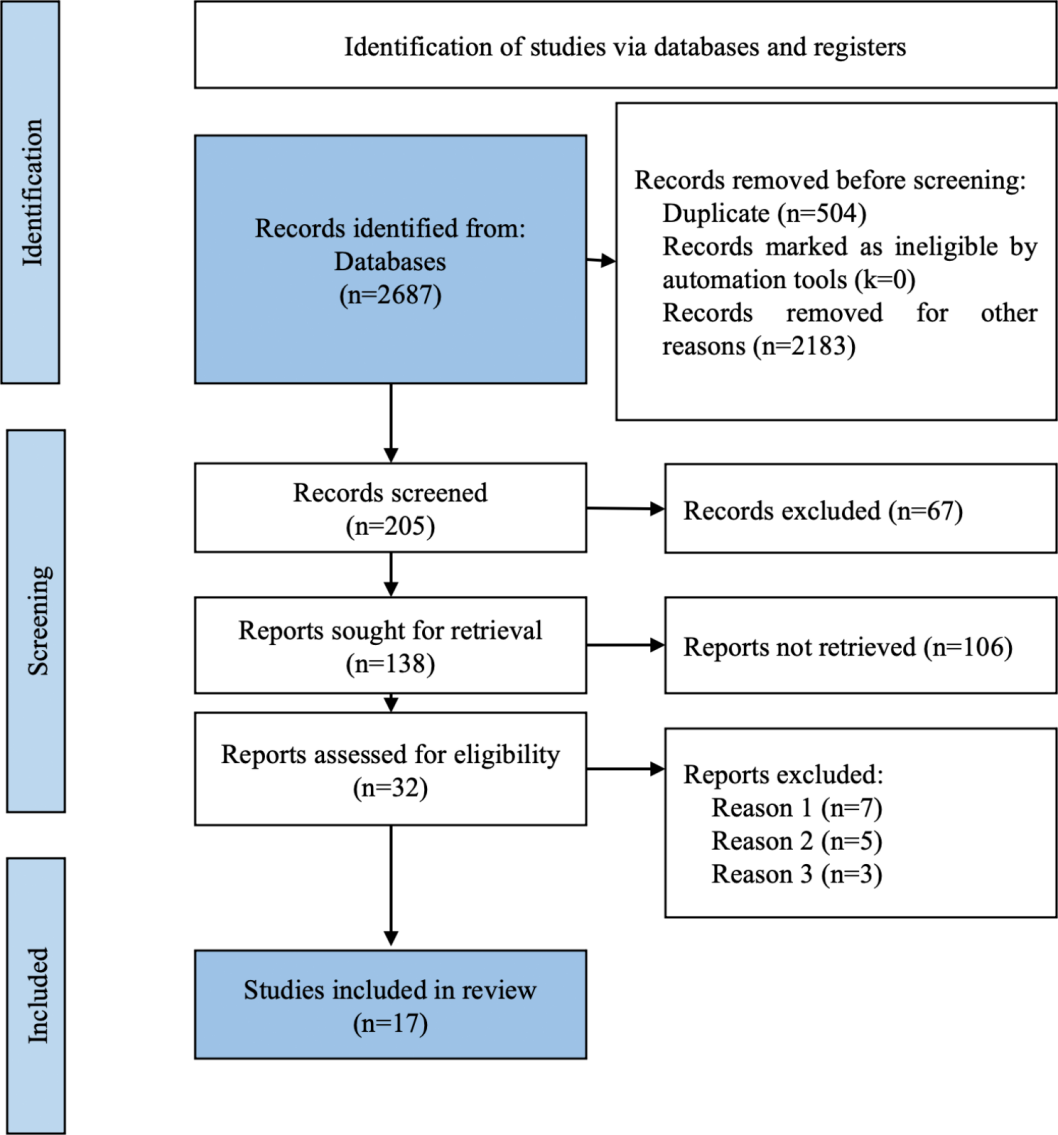
PRISMA flow chart

The search yielded 2,687 records, of which 205 were identified for full-text review (Fig. 1).

**Table 1:** Characteristics of included studies (n = 17)

Author, year, country	Study design	STROBE(21) Reporting Guidelines	Age of participants	No. Of subjects MetS+/MetS-	MetS criteria	Results						
Cheng, et al., 2017, Italy [24].	Cross sectional study	18	**Men** MetS + 56.57 ± 16.25 MetS- 47.88 ± 18.45 **Women** MetS + 56.61 ± 17.58 MetS- 44.57 ± 18.36	**Men** 969/2595 Total 3564 **Women** 1130/2676 Total 3806	NCEP ATP III	ALT values were significantly higher in MetS + participants.						
						**Men** MetS + 33.89 ± 23.55 (ALT) MetS- 29.3 ± 32.27 (ALT)				**Women** MetS + 24.92 ± 58.29 (ALT) MetS 20.45 ± 19.43 (ALT)		
Cheng YL, et al., 2017. Taiwan [25]	Cohort study	18	MetS + 56.3 ± 12.5 MetS- 50.6 ± 13.2	8564/21,233 Total 29,797	IDF	Subjects with MetS + have higher ALT, AST and GGT levels compared to subjects without MetS-.						
						MetS + 35.1 ± 29.1 (ALT) MetS- 23.8 ± 17.6 (ALT)		MetS + 26 ± 18.2 (AST) MetS- 21.9 ± 10.3(AST)				MetS + 34.2 ± 50.9 (GGT) MetS-21 ± 28.4 (GGT)
Choi, et al., 2017. Japan [26]	Cross sectional study	21	**Men** MetS + 49.5 ± 6.5 MetS- 48.8 ± 6.1	**Men** 251/474 Total 725	NCEP ATP III	ALT, AST, and GGT were significantly higher in middle-aged men with MetS + than in those without MetS-.						
						MetS + 37.5 ± 22.5 (ALT) MetS- 27.6 ± 17.7 (ALT)		MetS + 30.6 ± 12.4 (AST) MetS- 27.4 ± 13 (AST)				MetS + 58.3 ± 48.1 (GGT) MetS- 40.8 ± 49.2 (GGT)
Huang, et al., 2018. China [27]	Cross sectional study	18	**Men** MetS + 45.2 ± 9.4 MetS-42.14 ± 11.0 **Women** MetS + 48.5 ± 13.3 MetS- 41.9 ± 10.9	**Men** 43/176 Total 219 **Women** 18/196 Total 214	NCEP ATP III	Subjects with elevated ALT levels are at increased risk of MetS. ALT may be significantly associated with the presence of MetS.						
						**Men** MetS + 27.7 ± 7.5 (ALT) MetS- 24.4 ± 8.9 (ALT)				**Women** MetS + 22.3 ± 9.5 (ALT) MetS- 17.4 ± 7.6 (ALT)		
Kim, et al., 2022, Korea [28].	Cross sectional study	19	**Men** MetS + 68.5 ± 6.1 MetS- 69.5 ± 6.3 **Women** MetS + 69.3 ± 6.1 MetS- 68.8 ± 6.4	**Men** 583/1106 Total 1689 **Women** 1299/1493 Total 2792	Harmonised criteria	Elevated ALT and AST levels in MetS + subjects.						
						**Men** MetS + 25.8 ± 15.3 (ALT) MetS- 20.1 ± 10.3 (ALT) MetS + 25.2 ± 12.4 (AST) MetS- 23.3 ± 7.7 (AST)				**Women** MetS + 21.2 ± 12.3 (ALT) MetS-17.9 ± 11.3 (ALT) MetS + 23.3 ± 9.2 (AST) MetS- 22.5 ± 8.3 (AST)		
Kohsari et al., 2021, Iran [29].	Cross sectional study	18	Age of participants 47.3 ± 4.1	Men 1329/3397 Total 4730 Women 1936/3141 Total 5092	Harmonised criteria	Significant association between elevated ALT, AST, GGT and ALP levels and increased risk of MetS.						
						MetS + 27.6 ± 27.1 (ALT) MetS- 23.5 ± 13.9 (ALT)	MetS + 21.8 ± 8.5 (AST) MetS- 21.2 ± 8.9 (AST)					MetS + 28.9 ± 22.2 (GGT) MetS- 22.4 ± 18.2 (GGT)
Kuo et al., 2018, Taiwan [30].	Cross sectional study	19	MetS + 61.0 ± 11.0 MetS − 57.5 ± 11.6	54,361/125,998 Total 180,359	Harmonised criteria	Subjects with MetS + had higher ALT and AST levels.						
						MetS + 33.1 ± 25.0 (ALT) MetS- 24.6 ± 19.1 (ALT)			MetS + 28.6 ± 15.8 (AST) MetS- 25.1 ± 12.1(AST)			
Liu, et al., 2018. China [31]	Cross sectional study	19	MetS + 69.58 ± 7.01 MetS- 70.04 ± 7.65	524/920 Total 1444	Harmonised criteria	Elevated ALT, GGT and ALP levels are positively associated with the prevalence of MetS in the elderly population.						
						MetS + 26.98 ± 15.51 (ALT) MetS- 22.01 ± 12.58 (ALT)		MetS + 23.37 ± 8.85 (AST) MetS- 23.25 ± 8.66 (AST)				MetS + 29.80 ± 19.54 (GGT) MetS- 23.42 ± 18.93 (GGT)
Mitsuhashi et al., 2017, Japan [32].	Cohort study	18	MetS + 50.1 ± 9.3 MetS- 44.5 ± 9.4	698/13,266 Total 13,964	IDF	Higher AST, ALT and GGTP values in non-fatty liver MetS subjects.						
						MetS + 23.2 ± 12.7 (ALT) MetS- 17.5 ± 11.4 (ALT)		MetS + 20.7 ± 11.0 (AST) MetS- 17.8 ± 8. (AST)				MetS + 34.6 ± 40.0 (GGTP) MetS- 19.3 ± 19.0 (GGTP)
Osadnik et al., 2020, Poland. [33]	Cross sectional study	19	MetS + 28.07 ± 4.48 MetS- 26.86 ± 4.49	70/390 Total 460	Buscemi et al. (46)	MetS + subjects had increased activity of liver enzymes ALT, AST and GGTP.						
						MetS + 30.61 ± 26.97 (ALT) MetS- 18.74 ± 16.01 (ALT)	MetS + 31.06 ± 54.85 (AST) MetS- 20.58 ± 9.7 (AST)				MetS + 36.27 ± 38.78 (GGTP) MetS- 16.56 ± 9.65 (GGTP)	
Sakane et al., 2020, Japan [34].	Cluster randomised controlled trial	20	MetS + 49.4 ± 6.7 MetS- 48.4 ± 7.9	490/844 Total 1334	NCEP ATP III	MetS + group has elevated AST and ALT levels compared to the MetS- group.						
						MetS + 27.9 ± 11.4 (AST) MetS- 23.4 ± 13.8 (AST)			MetS + 37.2 ± 22.0 (ALT) MetS- 25 ± 15.8 (ALT)			
Sobage et al., 2020, Japan [35].	Cross sectional study	20	MetS + 51.2 ± 9.7 MetS- 55.4 ± 7.2	418/2246 Total 2664	NCEP ATP III	ALT, AST, GGT and the prevalence of NAFLD were significantly higher in the MetS + group.						
						MetS + 37.0 ± 25.3 (ALT) MetS- 19.3 ± 11.4 (ALT)		MetS + 29.6 ± 15.1 (AST) MetS- 22.3 ± 6.6 (AST)				MetS + 48.0 ± 37.5 (GGTP) MetS- 26.2 ± 27.0 (GGTP)
Sumiyoshi et al., 2018, Japan [36].	Retrospect. observational study	19	MetS + 50.8 ± 9.5 MetS- 48.8 ± 9.6	1031/13,762 Total 14,793	Japan Diagnostic criteria	Higher ALT and AST levels are observed in the MetS + group.						
						MetS + 32 ± 22 (ALT) MetS- 21 ± 15 (ALT)			MetS + 25 ± 12 (AST) MetS- 21 ± 9 (AST)			
Wang, et al., 2018, China [37].	Cross sectional study	19	MetS + 69.34 ± 7.11 MetS- 70.6 ± 6.76	161/307 Total 468	Harmonized criteria	Significantly higher ALT levels in the MetS + group.						
						MetS + 24.77 ± 14.58 (ALT) MetS- 21.64 ± 14.17 (ALT)			MetS + 24.62 ± 14.58 (AST) MetS- 24.19 ± 8.80 (AST)			
Wang, et al., 2020, China [38].	Cross sectional study	19	MetS 68.79 ± 6.53 MetS- 68.34 ± 6.58	2207/1791 Total 3998	NCEP ATP III	The combined increase in serum uric acid (SUA) and alanine aminotransferase (ALT) were significantly correlated with MetS and its components.						
						MetS + 22.32 ± 18.39 (ALT) MetS- 18.27 ± 13.52 (ALT)			MetS + 23.18 ± 14.31 (AST) MetS- 22.08 ± 10.98 (AST)			
Wu et al., 2021, Taiwan [39].	Prospective Cohort study	20	MetS + 42.88 ± 8.96 MetS- 37.97 ± 9.0	66/680 Total 746	NCEP ATP III	Higher ALT and AST levels are associated with an elevated risk of MetS+.						
						MetS + 31.77 ± 23.77 (ALT) MetS- 20.58 ± 24.07 (ALT)			MetS + 22.92 ± 12.09 (AST) MetS- 19.49 ± 12.52 (AST)			
Yang, et al., 2021. China [40]	Case-control study	18	MetS + 54.89 ± 12.53 MetS- 45.67 ± 12.73	538/5164 Total 5702	Chinese society of Diabetes	Higher ALT, AST levels in MetS + subjects.						
						MetS- 30.19 ± 19.87 (ALT) MetS- 25.38 ± 20.74 (ALT)			MetS + 25.65 ± 10.82 (AST) MetS- 23.37 ± 11.42 (AST)			

Abbreviations: ALP, alkaline phosphatase; ALT, alanine aminotransferase; AST, aspartate aminotransferase; GGT, gamma-glutamyltransferase; IDF, International Diabetes Federation; MetS, metabolic syndrome; NAFLD, Non-alcoholic fatty liver disease; NCEP ATP III, National Cholesterol Education Program Adult Treatment Panel III; STROBE, Strengthening the Reporting of Observational Studies in Epidemiology; SUA, serum uric acid

Of these, 17 met the inclusion criteria and were selected for systematic review and meta-analysis. Cohen’s Kappa clinical concordance index between the two authors (E.R.C and M.R.S.) who conducted the search was 82.8% (95% CI 70.3–95.3).

The detailed characteristics of the selected studies are shown in Table 1.

In total, 17 articles compared liver enzyme concentrations between 76,686 MetS + and 201,855 MetS- subjects. The age of the participants ranged from 22 to 78 years. Most papers (82.35%) [25, 26, 29–40] included participants of both sexes but analysed the data globally; 3 studies (17.65%) [24, 27, 28] collected data from men and women separately. Concerning origin, 5 articles were developed in China [27, 31, 37, 38, 40], 5 in Japan [26, 32, 34, 35, 37], three articles in Taiwan [25, 30, 39], 1 in Italy [24], 1 in Poland [33], Korea [28] and Iran [29]. Data were extracted from 17 reports from ALT [24–40], 15 studies from AST [25, 26, 28–40], and five from GGT [25, 26, 29, 31, 32].

In seven of the manuscripts [24, 26, 27, 34, 35, 38, 39], MetS was defined according to the criteria of the third report of the National Cholesterol Education Program (NCEP - ATP III) [41]; 5 studies [28–31, 37] assessed MetS using the harmonised criteria [42]; 2 papers [25, 32] using the International Diabetes Federation (IDF) definition [43]; and one article [40] used the Chinese diabetes Society criteria [44]. Finally, Sumiyoshi et al. [36] used the Japanese standards [45] and Osadnik [33], those defined by Buscemi et al. [46].

### Methodological quality assessment

According to the STROBE reporting guidelines [21], all reports scored 18 points or more out of the 22 items included (highest tercile). No articles were excluded for poor methodological quality. The score for each of the papers is shown in Table 1.

### Bias risk analysis

**Fig. 2:**
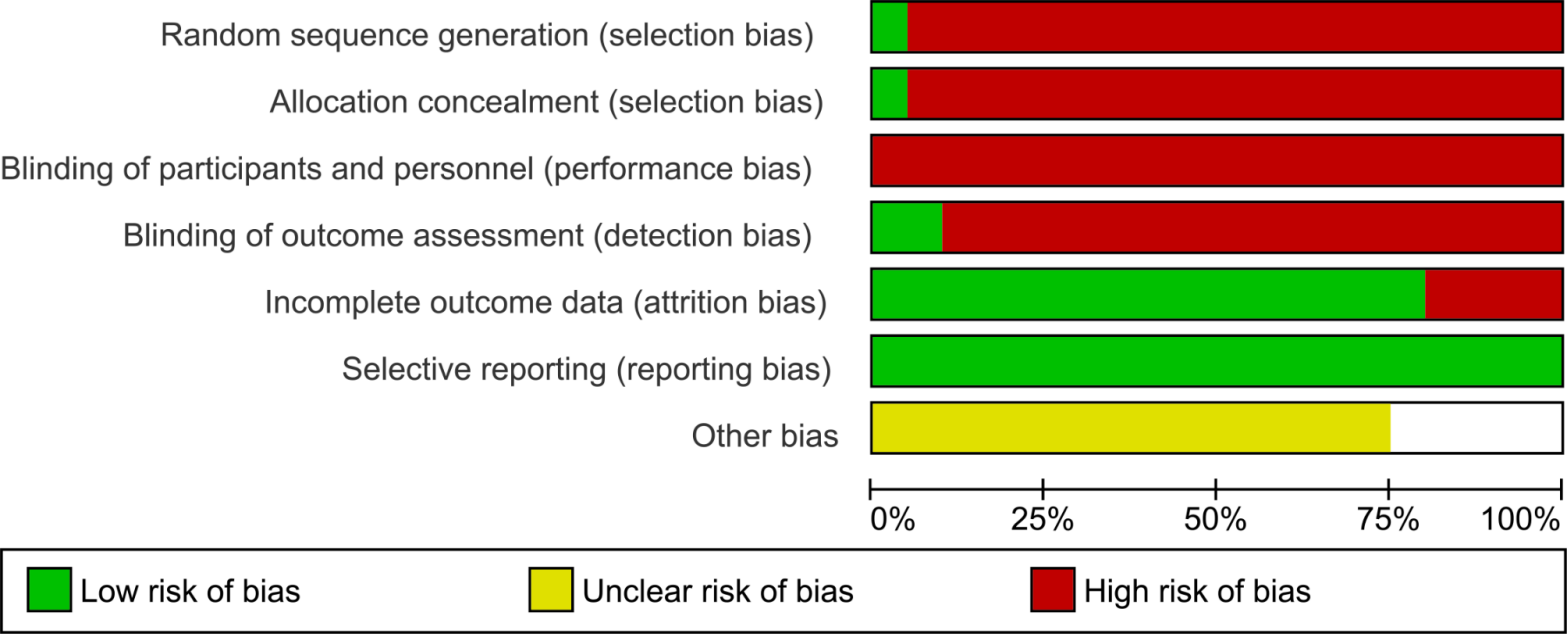
The overall risk of bias in the studies

**Fig. 3:**
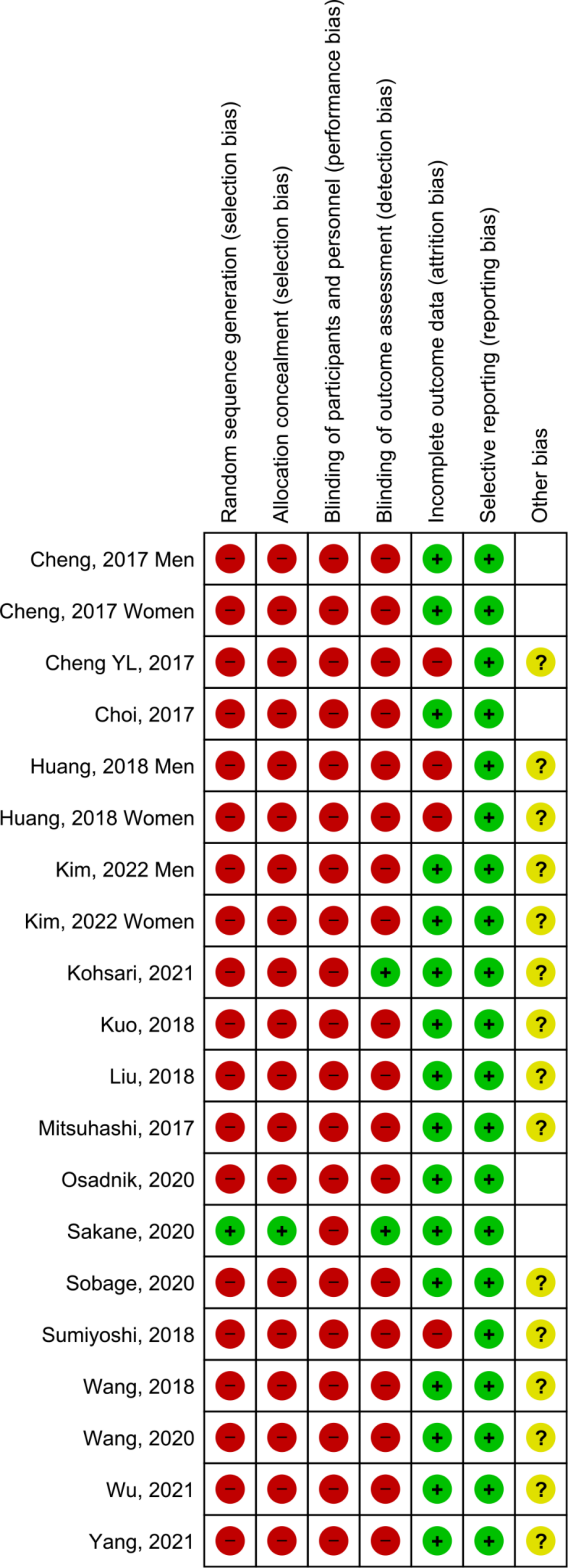
Summary of risk of bias by study

Overall (Fig. 2), it can be seen that the main biases were: random sequential generation, concealment of allocation and blinding of outcome evaluation (related to participants and staff). Figure 3 represents the individual assessment of the included studies.

### Quantitative analysis. Meta-analysis

**Fig. 4:**
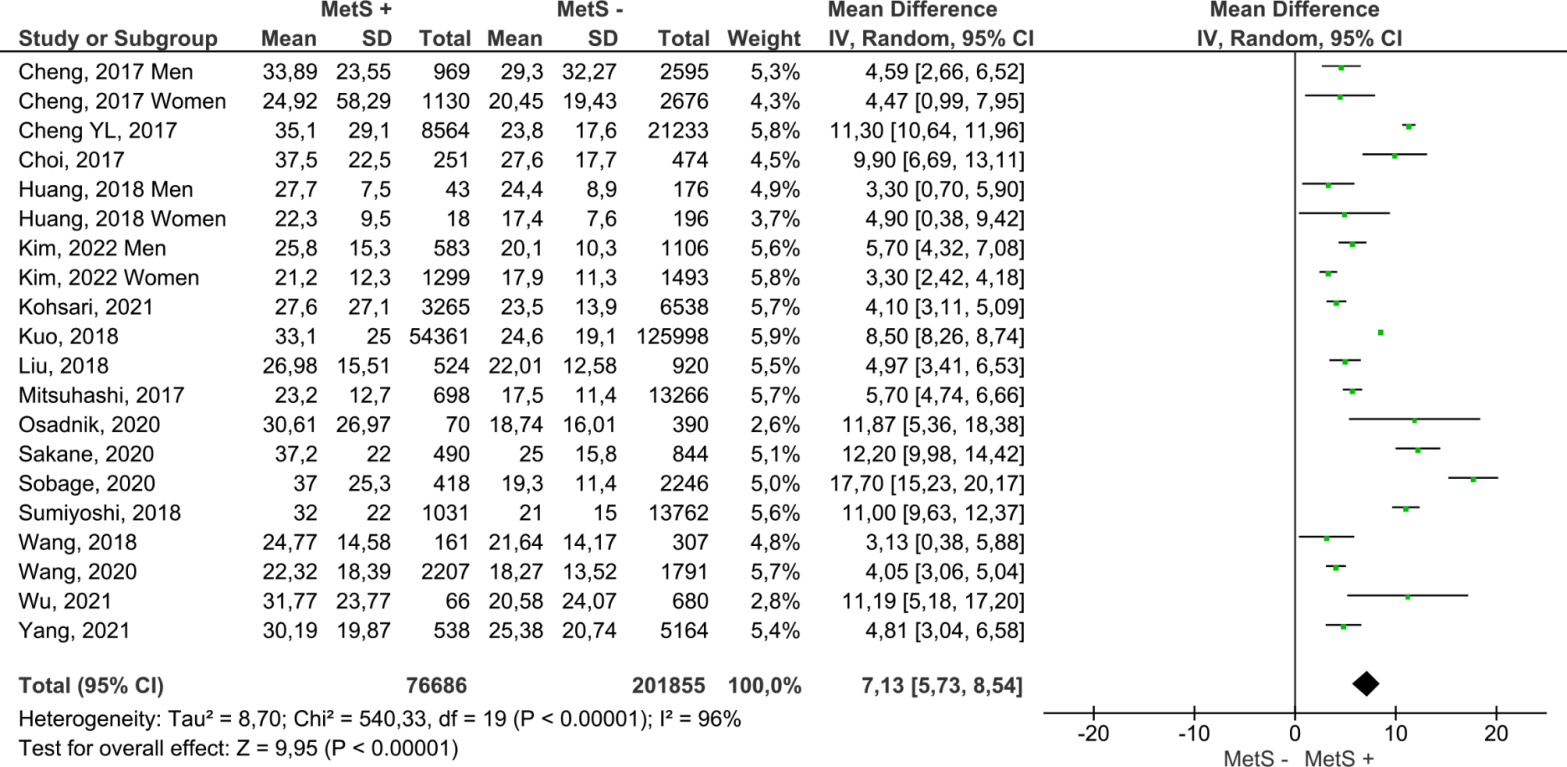
Results and summary statistics of studies analysing ALT levels in the total population with and without metabolic syndrome (MetS)

**Fig. 5:**
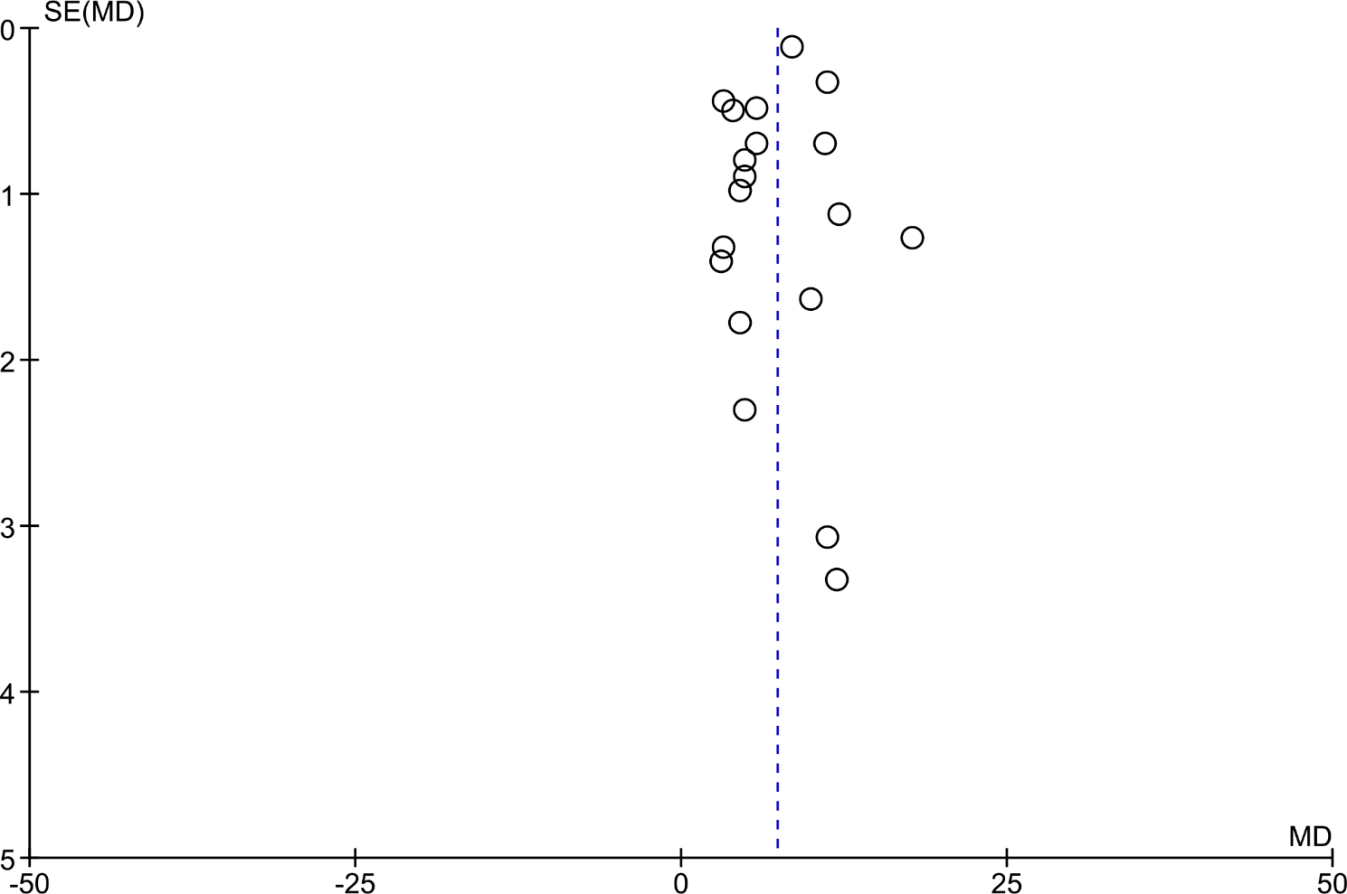
Publication bias ALT (Funnel plot)

**Fig. 6:**
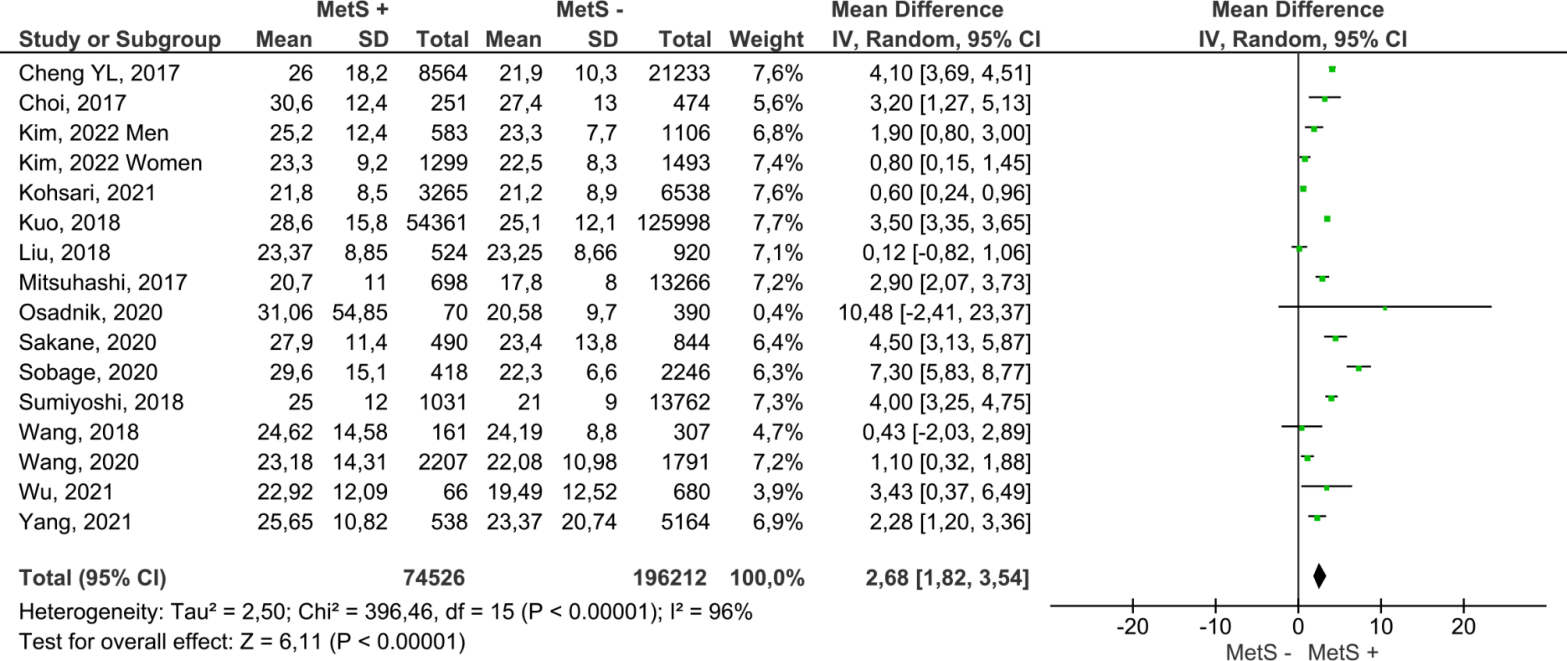
Results and summary statistics of studies analysing AST levels in the total population with and without metabolic syndrome (MetS)

**Fig. 7:**
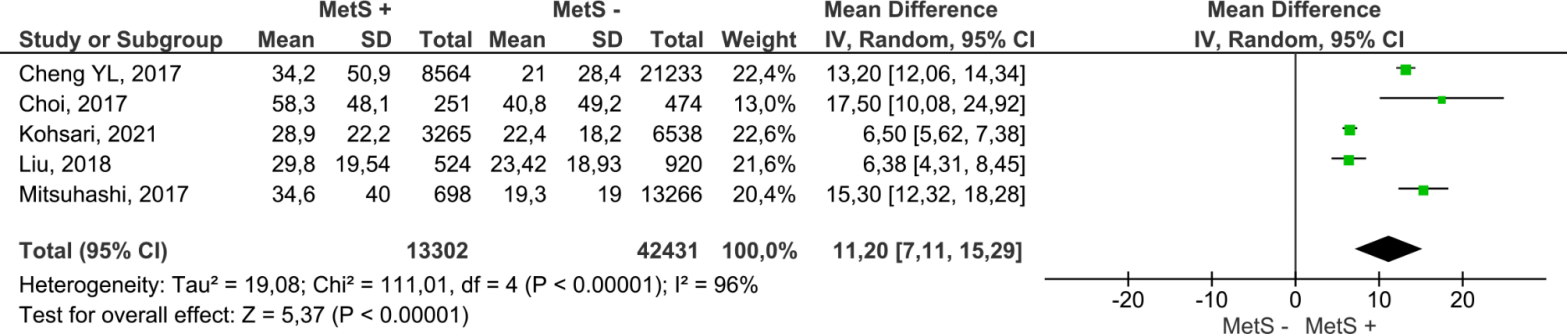
Results and summary statistics of studies analysing GGT levels in the total population with and without metabolic syndrome (MetS)

Figure 4 includes the results for both sexes from the 17 reviewed papers. MetS + subjects showed a higher mean ALT, with the difference reaching 7.13 IU/L (95% CI 5.73–8.54); compared to MetS- subjects. Furthermore, this analysis had a low risk of publication bias (Fig. 5). On the other hand, MetS + subjects showed a higher mean AST, namely, the mean difference was 2.68 IU/L (95% CI 1.82–3.54); compared to MetS- subjects (Fig. 6). Concerning GGT (Fig. 7), the mean difference reached 11.20 IU/L (95% CI 7.11–15.26), being higher among MetS + subjects. All results showed considerable heterogeneity (> 95%). Annex I shows a low risk of publication bias in the AST and GGT analysis.

### Quality of evidence

Using the Grade Pro tool, the quality of the evidence in this meta-analysis was assessed, and a very low degree of certainty was obtained due to the high inconsistency and risk of bias in the included studies (Table 2).

**Table 2:** Evidence profile with GRADE pro for the meta-analyses

Certainty assessment							No. of subjects		Size of the effect	Quality of evidence
No. of studies	Study design	Risk of bias	Inconsistency	Indirect evidence	Imprecision	Other considerations	MetS+	MetS-	Mean difference (95% CI)	
**ALT Meta-analysis**										
n = 17	Observational studies	serious	Very serious	It is not serious	It is not serious	dose-response gradient	76.686	201.855	7.13 (5.73–8.54)	⨁◯◯◯ Very low
**AST Meta-analysis**										
n = 15	Observational studies	serious	Very serious	It is not serious	It is not serious	dose-response gradient	74.526	196.212	2.68 (1.82–3.54)	⨁◯◯◯ Very low
**GGT Meta-analysis**										
n = 5	Observational studies	serious	Very serious	It is not serious	It is not serious	dose-response gradient	13.302	42.431	11.2 (7.11–15.29)	⨁◯◯◯ Very low

Abbreviations; ALT, alanine aminotransferase; AST, aspartate aminotransferase; GGT, gamma-glutamyltransferase; MetS, metabolic syndrome; CI, confidence interval

## Discussion

This systematic review with meta-analysis was conducted to analyse the most recent evidence on the relationship between MetS and liver enzymes (ALT, AST and GGT). Seventeen articles were selected in which the effect size was quantified and the limitations that have conditioned the results of the different studies. All demonstrated sufficient reliability and methodological quality regarding the association between ALT, AST, GGT and MetS.

The present meta-analysis has shown the relationship between the levels of different liver enzymes studied and MetS. The concentration of the liver enzymes studied in the 76,686 MetS + subjects was significantly higher than in the group of 201,855 controls (MetS-).

The presence of elevated liver enzymes may precede the development of MetS, with alterations of the liver being observed that are directly related to metabolic problems, such as NAFLD. Recently, it was considered a manifestation of metabolic diseases. However, it has been suggested that NAFLD temporarily precedes DM and that hepatic steatosis may cause insulin resistance [47] and may be an early sign of the development of metabolic diseases [48]. In addition, when fat is deposited in insulin-sensitive organs such as the liver, muscle and visceral compartments, free fatty acids and inflammatory cytokines increase while adiponectin levels decrease [49, 50]. These changes can lead to peripheral insulin resistance, early atherogenesis, impaired glucose metabolism and MetS [51, 52].

Previous studies have reported that NAFLD predates MetS components such as impaired fasting glucose and hypertension [53–55]. The study in young adults by Yoo et al. [56] concludes that the degree of hepatic steatosis can predict the future occurrence of MetS. Several studies have reported that NAFLD contributes to the development of DM2 and is associated with increased cardiovascular risk [57, 58]. The meta-analysis of prospective studies by Ballestri et al. [59] concluded that NAFLD significantly increases the incident risk of DM2 and MetS. This fact is highly relevant given that NAFLD is associated with elevated liver enzymes, such as ALT, AST and GGT, so early detection can help in interventions to prevent metabolic diseases such as MetS.

Concerning MetS, studies have shown that liver enzymes could be new candidate biomarkers for its early diagnosis. Our results are consistent with the associations reported between liver enzymes and MetS by other authors. The cross-sectional study by Chen et al. [17] concludes that serum ALT levels, even within the reference range, are significantly associated with MetS. The study by Sattar et al. [60] informs that serum ALT levels, but not AST levels, increased progressively as the number of MetS components increased. The meta-analysis of 10 prospective cohort studies by Kunutsor et al. [61] reported a dose-response relationship between GGT level and the risk of MetS. The meta-analysis by Liu et al. [62], involving 9 cohort studies, evidenced a positive association between GGT levels and the risk of MetS independent of alcohol intake.

In addition, there are significant gender differences, with males having higher levels than females, and the reference ranges established by the laboratories also vary. The study by Cheng et al. [24] reveals that male subjects had a higher prevalence of MetS and higher ALT levels; these results are in line with studies by Huang et al. [27] and Kim et al. [28].

However, further epidemiological investigations using longitudinal designs are needed to understand the associations between serum ALT, AST, and GGT levels and MetS.

These findings have important clinical implications regarding the optimal strategies to be adopted to prevent the development of MetS. In addition, monitoring liver enzyme values to detect their gradual elevation could alert to future metabolic problems.

### Limitations and strengths

At the methodological level, the assessment of risks of bias in studies is a major issue in this type of research, in line with PRISMA recommendations. Studies with similar methodologies but with discrepancies in quality may have biased results. The quality of the evidence obtained is “very low” because observational studies have been analysed where there is a high risk of bias and, in addition, present a very high inconsistency (heterogeneity).

The authors were unable to fully examine the impact of adjustment for all known and potential risk factors, due to the varying degree of adjustment for confounding factors across individual studies.

One of the main strengths of this review is the comprehensive search covering a wide geographical area. In addition, a large sample size of subjects with and without MetS was included, which increased the study’s statistical power. However, considering some limitations, interpreting the findings in this systematic review and meta-analysis should be done cautiously. Firstly, non-randomised comparisons in observational studies may suffer from bias, which could affect the findings and thus weaken the strength of the evidence. Secondly, the included studies used different definitions to diagnose MetS, which may alter the findings. Also, the heterogeneity of the analyses was very high, which makes the results less robust. Finally, another limitation was that no additional strategies were used in the current search to locate unpublished reviews (grey literature).

## Conclusions

The results have shown that MetS + subjects have higher levels of all liver enzymes tested than MetS- subjects. These findings provide a rationale for further evaluation of the relationship of liver enzymes in the pathophysiological process of MetS and could lead to new perspectives in early diagnosis.

The relevance of these findings has several implications for clinical practice, such as early diagnosis of MetS, early prevention of associated liver damage, better understanding of the pathophysiology, as well as the management and direction of effective care strategies for these patients.

However, primary studies with higher methodological quality should be performed to provide more robustness to the collected findings. Also, regarding this severe health problem, more research is needed in different populations to identify the importance of liver enzymes in MetS or other cardiovascular diseases.

### Electronic supplementary material

Below is the link to the electronic supplementary material.

Supplementary Material 1

Supplementary Material 2

Supplementary Material 3

## Data Availability

All data generated or analysed during this study are included in this published article [and its supplementary information files].
