# Supplementary figures and images for: Metabolic syndrome and transaminases: systematic review and meta-analysis
Source: Diabetol Metab Syndr. 2023 Oct 30;15:220. doi: 10.1186/s13098-023-01200-z (PMC10614379; doi:10.1186/s13098-023-01200-z)

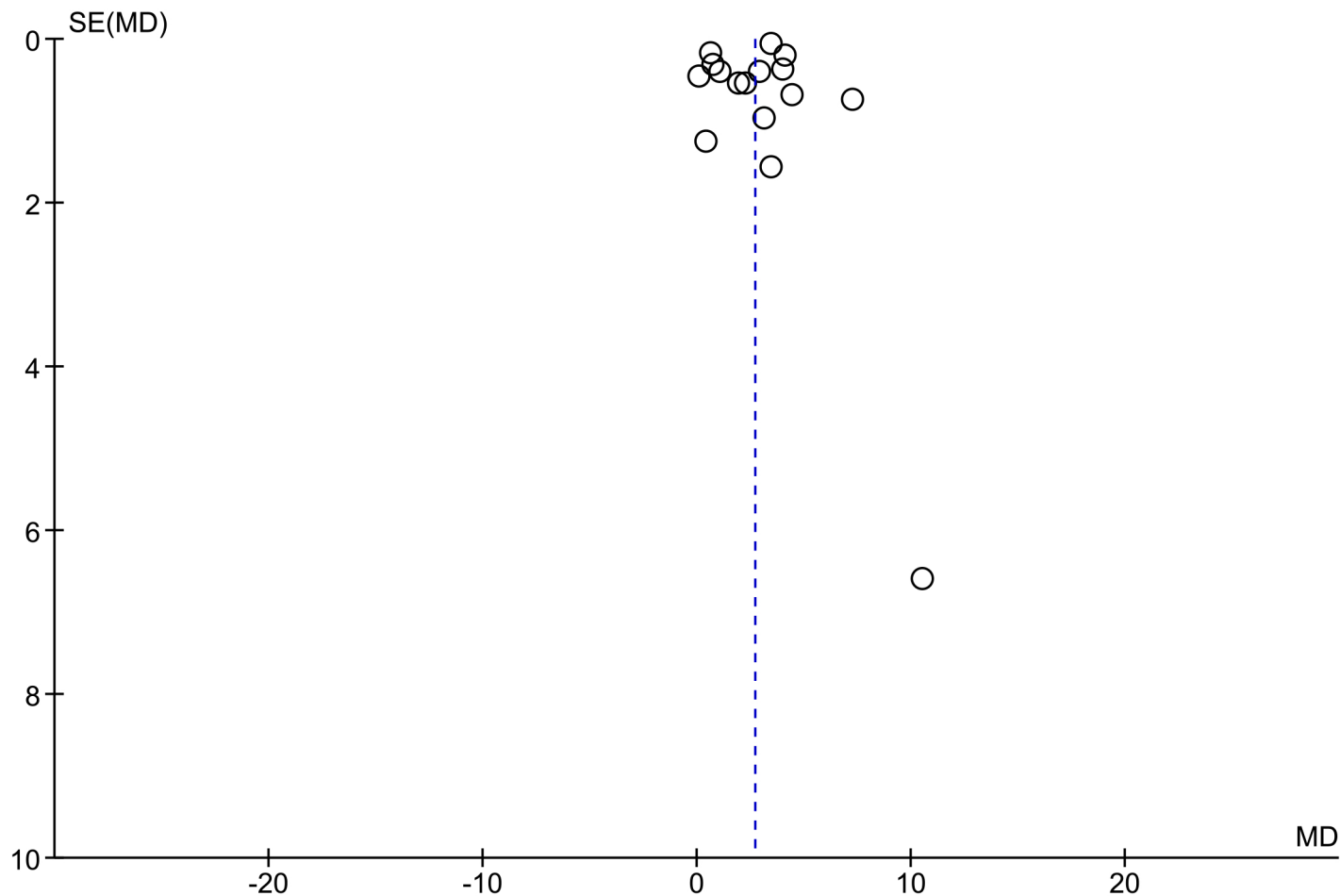

Fig 1. Publication bias AST (Funnel plot).

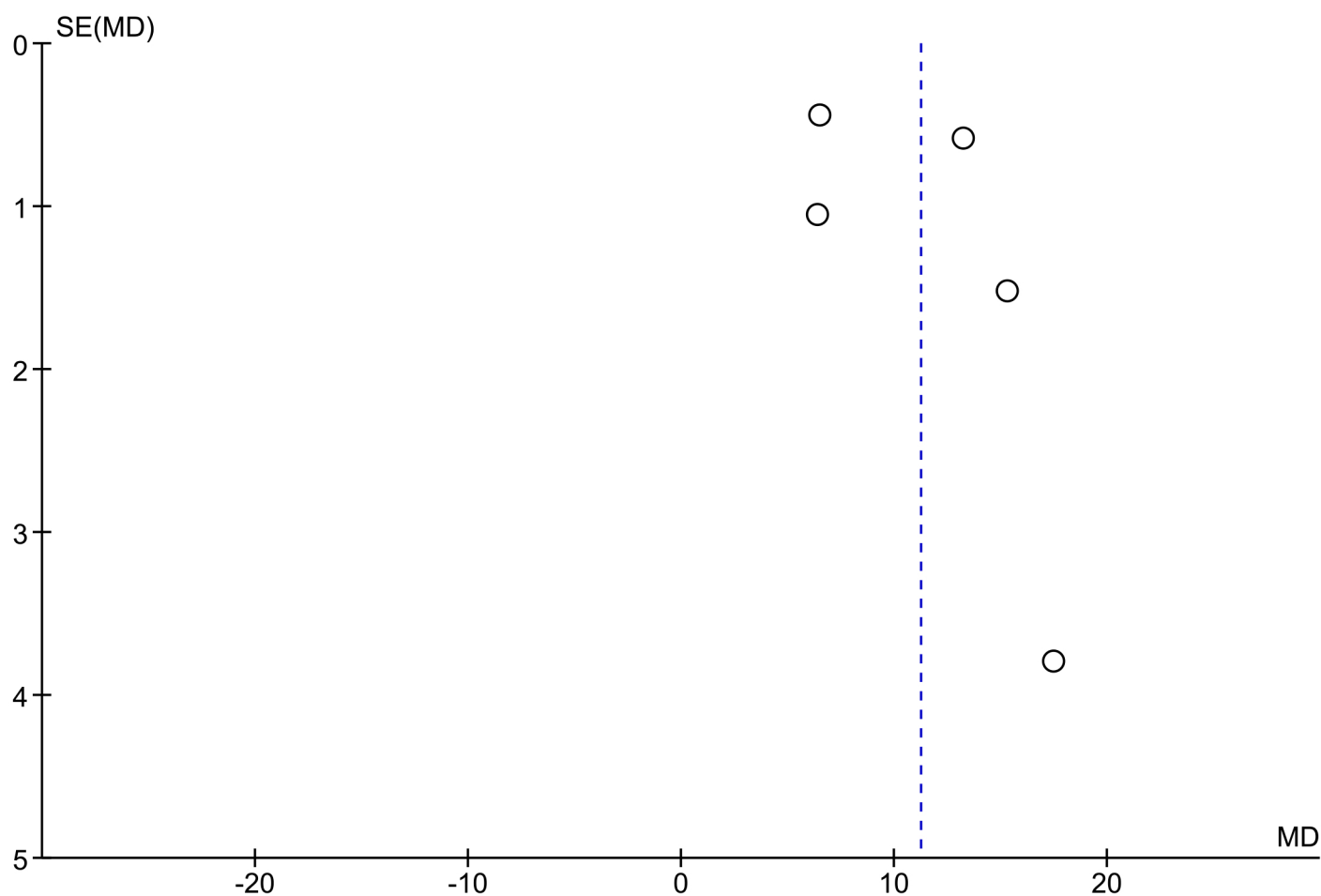

Fig 2. Publication bias GGT (Funnel plot).

Supplement: Supplementary file 1 — Supplementary Material 1 [file 13098_2023_1200_MOESM1_ESM.pdf]
