# Supplementary material for: Metabolic syndrome and transaminases: systematic review and meta-analysis
Source: Diabetol Metab Syndr. 2023 Oct 30;15:220. doi: 10.1186/s13098-023-01200-z (PMC10614379; doi:10.1186/s13098-023-01200-z)
Supplement: Supplementary file 3 — Supplementary Material 3 [file 13098_2023_1200_MOESM3_ESM.docx]

**ELECTRONIC SEARCH STRATEGY**

The search strategy used in PubMed is detailed below:

| Search | Search strategy | No. of papers |
| --- | --- | --- |
| #1 | (metabolic syndrome) AND (alanine transaminase) Filters: from 2017-2022 | 473 |
| #2 | (metabolic syndrome) AND (aspartate aminotransferase) Filters: from 2017-2022 | 271 |
| #3 | (metabolic syndrome) AND (gamma-Glutamyltransferase) Filters: from 2017-2022 | 96 |

((("metabolic syndrome"[MeSH Terms] OR ("metabolic"[All Fields] AND "syndrome"[All Fields]) OR "metabolic syndrome"[All Fields]) AND ("alanine transaminase"[MeSH Terms] OR ("alanine"[All Fields] AND "transaminase"[All Fields]) OR "alanine transaminase"[All Fields])) OR (("metabolic syndrome"[MeSH Terms] OR ("metabolic"[All Fields] AND "syndrome"[All Fields]) OR "metabolic syndrome"[All Fields]) AND ("aspartate aminotransferases"[MeSH Terms] OR ("aspartate"[All Fields] AND "aminotransferases"[All Fields]) OR "aspartate aminotransferases"[All Fields] OR ("aspartate"[All Fields] AND "aminotransferase"[All Fields]) OR "aspartate aminotransferase"[All Fields])) OR (("metabolic syndrome"[MeSH Terms] OR ("metabolic"[All Fields] AND "syndrome"[All Fields]) OR "metabolic syndrome"[All Fields]) AND ("gamma glutamyltransferase"[MeSH Terms] OR "gamma glutamyltransferase"[All Fields] OR ("gamma"[All Fields] AND "glutamyltransferase"[All Fields]) OR "gamma glutamyltransferase"[All Fields]))) AND (y_5[Filter])
